# Measuring Responsible Gambling amongst Players: Development of the Positive Play Scale

**DOI:** 10.3389/fpsyg.2017.00227

**Published:** 2017-02-23

**Authors:** Richard T. A. Wood, Michael J. A. Wohl, Nassim Tabri, Kahlil Philander

**Affiliations:** ^1^GamRes LimitedHawkesbury, ON, Canada; ^2^Gambling Lab, Psychology, Carleton UniversityOttawa, ON, Canada; ^3^British Columbia Lottery Corporation, Responsible GamblingVancouver, BC, Canada

**Keywords:** responsible gambling, harm reduction, harm avoidance, gambling disorder, corporate social responsibility

## Abstract

The modern gambling industry has, by-in-large, assumed a duty of care to minimize the risks associated with gambling, which has manifested in responsible gambling (RG) programming (e.g., educating players about the odds of success). The current study fills a void in gambling operators, regulators, and researchers ability to measure RG beliefs and behavior in their player-base, with the development and validation of the Positive Play Scale (PPS). In Study 1, we reviewed the literature and consulted 30 players as well as 10 RG experts to help generate a definition of RG beliefs and behavior that helped guide item generation. In Study 2, regular players (*N* = 1,551) of a Canadian provincial gambling operator completed a positive play survey. Four components from a principal components analysis (PCA) were extracted: Honesty and Control, Pre-commitment, Personal Responsibility, and Gambling Literacy. The PPS subscales were either not associated with gambling frequency or had small-to-moderate negative relationships with frequency of play for games most often associated with disordered gambling (e.g., electronic games). In Study 3 (*N* = 413), the factor structure of the PPS was confirmed and refined in a new sample of players. Moreover, a 1-month follow-up session demonstrated that the PPS has high test-retest reliability. The PPS is the first validated scale that reliably assesses the extent to which a consumer base has positive beliefs about gambling and gambles in a positive manner. The PPS can be used by the gambling industry to objectively assess the efficacy of their RG strategy, pinpoint specific areas for future focus, as well as examine the utility of new RG initiatives that aim to promote healthy patterns of gambling consumption. Furthermore, by examining the PPS scores for different player segments (e.g., sex, age, games played) it becomes possible to tailor RG strategy to the needs of specific players. In this way, RG strategy can be optimized by focusing resources where they will be most effective.

## Introduction

In industries with products that have the potential to harm to some customers' health, firms often become the focus of policymakers and other stakeholders, as those parties develop frameworks to minimize negative health related outcomes (Moodie et al., 2013). As public health goals tend to focus on population-wide outcomes, individual firm contributions may be difficult to reconcile due to the complexity of dynamics between products, individuals, and environments. Over the last 10 years, much of the gambling industry has accepted promotion of responsible gambling (RG) to their customers (Blaszczynski et al., 2004) to reduce harms caused by excessive gambling. The framework stipulates that although the consumer holds the ultimate responsibility for their playing behavior, the gambling industry holds a duty of care.

Indeed, an ethical gambling operator should institute a program that assists players to make well-informed choices about their gambling behavior, in order that they may gamble in accordance with personally affordable money and time limits (see Blaszczynski et al., 2011). This strategy is important, because a significant number of gambling players believe they can exert control over the outcome of objectively uncontrollable gambling games, have inflated beliefs about their chances of winning, and spend more money and time than they can afford—factors that can lead to excessive gambling (Blaszczynski and Nower, 2007; Hodgins and Holub, 2007). In addition, gambling operators can have positive impacts on players' well-being by deploying many tactics as part of the consumer experience (for a review see Wohl et al., 2014b).

Among other tactics, tools have been created that inform players about the odds of winning (Wohl et al., 2010; Blaszczynski et al., 2014), describe the benefits of limit setting and adherence (Auer and Griffiths, 2013; Stewart and Wohl, 2013; Kim et al., 2014), and provide personalized player feedback (i.e., personalized communications about engagement in risky gambling behaviors; Wood and Wohl, 2015). These and other tools have become an inherent part of many gambling operators' harm minimization strategies, in part, because the research community has demonstrated their RG utility—they can help players to decide upon suitable spending limits and help them to stay within those limits—and as such provide players with a more ethical and less harmful product (Wood et al., 2014).

Nevertheless, one critical aspect for evaluating gambling industry RG initiatives that have been largely overlooked is the examination of the extent to which players in a given jurisdiction actually engage in RG. Moreover, gambling operators currently have little or no knowledge about the extent to which their customers have positive (i.e., accurate and realistic) beliefs about their gambling, or the nature and extent to which their players engage in positive, responsible behaviors. As such, gambling operators must develop their overall RG programs based largely on RG theory or evaluations of individual RG initiatives, with little or no direct information about their specific player base. Gambling operators currently have no means to benchmark levels of RG beliefs and behaviors prior to implementing their RG program or across firms/jurisdictions, making it difficult to evaluate whether an RG program positively contributed to wider health framework related goals.

The closest approximation for identifying and measuring positive, responsible gambling beliefs and behaviors in a player base, has been through identifying the extent to which customers report symptoms of disordered gambling (i.e., a high-score on a disordered gambling screen) and then deducing that those who do not report symptoms are playing responsibly. However, disordered gambling is an issue that affects a very small minority of the overall population (between 0.1 and 5.8% world-wide based on past-year gambling, Calado and Griffiths, 2016). As such, a focus on disordered players is not particularly informative about the vast majority of players who do not exhibit measurable problems. Additionally, some researchers have argued that RG is not merely the absence of disordered gambling, but also involves the presence of positive gambling elements (e.g., considering a budget prior to gambling, playing for fun, balancing gambling with other leisure interests; see Wood and Griffiths, 2015; Hing et al., 2016). Conversely, some players may play “irresponsibly” and hold erroneous beliefs about gambling, but not exhibit any signs of a gambling problem (at least at the time of measurement). As such, we argue that RG is best identified and measured via the presence of positive gambling beliefs and behaviors (i.e., positive play).

The current research was designed to develop and validate the positive play scale (PPS)—a scale that could objectively identify and measure the extent of responsible play within a sample of players. The use of such a scale by the gambling industry would provide important information concerning the majority of players' beliefs and behaviors about RG. Moreover, it could help identify the utility of a particular RG strategy, aiding future RG strategy optimization. That is, areas of RG where players are scoring relatively low on the PPS, could be the focus for future RG strategy, whereas areas of RG that are scoring relatively high on the PPS would not require the provision of additional resources. Furthermore, by examining player segments (e.g., sex, age, games played) RG resources can be further applied to support those specific players who are found to score lowest. Additionally, concrete evidence for an individual firm's or overall industry's contribution to public health goals is objectively demonstrated. This is because using a validated RG scale before the introduction of a new RG strategy or initiative provides a benchmark level of RG in a population to compare both over time and between jurisdictions.

## Overview of the current research

Three studies were conducted. In Study 1, the aim was to generate the items that would form the PPS. To this end, we reviewed the existing RG literature and consulted relevant players as well as RG experts. Based on this, it was anticipated that positive play would be multidimensional, consisting of both positive beliefs (e.g., believing that the outcome of each game cannot be pre-determined) and positive behaviors (e.g., setting a money and time limit of gambling). As such, items were developed to measure both positive play beliefs and behaviors. This procedure enabled us to generate questionnaire items with both high content and face validity based on input from experts and players (see Sartori, 2010). In Study 2, the aim was to examine the psychometric properties of the PPS items and to pursue scale formation via principal components analysis (PCA). We also assessed the construct validity of the PPS through associations with self-reports of gambling frequency, disordered gambling severity, and disordered gambling beliefs (see Sartori and Pasini, 2007). The purpose of Study 3 was to replicate the factor structure of the PPS and to assess its reliability over time (1 month). Another aim of Study 3 was to further assess the validity of the PPS through associations with constructs that have been previously associated with gambling. These constructs were the Big-Five personality traits, impulsivity, general self-efficacy, financial dissatisfaction, and financially focused self-concept.

## Study 1: operationalizing and measuring responsible gambling

Before developing items for a scale that assesses positive play, we thought it prudent to operationally define responsible gambling in terms of beliefs and behaviors that encouraged amongst players. Current definitions of RG are sparse. The most often cited model of RG is the “Reno Model” (Blaszczynski et al., 2004), which focuses on the actions of gaming operators to assist players to help themselves in making well-informed choices. However, whilst the definition utilized by the model has been extremely useful for considering the design and implementation of RG strategy, it does not define what RG looks like from the player's perspective. That is, how should players think and behave to be considered “responsible?” This is important, because in order to measure the success of something it is necessary to have a clear notion of what “success” actually looks like. The lack of input from the player's perspective is a limitation of many current RG initiatives. Indeed, Wohl et al. (2014a) showed that when the player's perspective and feedback are incorporated into a RG tool, effectiveness is significantly enhanced. A more recent attempt to define RG, suggests that “the pivotal definition underpinning all RG policies and strategies: are initiatives that are designed to limit gambling expenditures to personally affordable levels” (Ladouceur et al., 2016, p. 3). However, this definition is again focused on policies and strategies and does not consider the consumer experience or how adherence to RG might be measured within a player sample.

In order to set parameters for item construction, we sought the input of 10 other experts in the field of gambling studies about the construct under investigation—RG. With the PPS, we wanted to go beyond defining what gambling operators/regulators/policymakers should do to promote RG. Instead, we wanted to understand how players might be expected to behave and think in relation to their gambling for it to be considered “responsible.” By doing so, a clearer notion of what RG “success” looks like can be achieved and measured.

To generate a new working definition of RG, we conducted a rapid evidence assessment of the RG literature, spoke to 30 players about RG, and consulted with 10 experienced researchers in the field (from five different countries). Based on these activities, the following working definition of RG was generated:

“RG, is when a player undertakes positive playing experiences and holds attitudes and beliefs that do not put them at risk for developing gambling problems. More specifically, this means only spending what is affordable to lose and sticking to personally allocated spend and time limits (formal or informal). Responsible play includes honesty and openness with self and others about personal gambling habits. Belief in luck or other superstitions may be present, but they do not have a significant negative impact on play. There is recognition that gambling will always involve some degree of chance.”

### Item selection

We developed an initial list of 61 potential items after reviewing the outcome variables used in RG-oriented research and in light of our working definition of RG. These items were sent back to the 10 experienced researchers in the field for feedback on possible re-wording and culling. Based on feedback, 11 items were culled. These 40-items were then presented to 30 players (male = 15, female = 15) who regularly played a wide range of gambling type games, for feedback on item clarity. As a result, some items were revised further and seven items culled, leaving 33 items (belief items = 20, behavior items = 13; see Tables 1, 2, respectively).

**Table 1:** **Rotated factor loadings from a principal component analysis of the PPS behavior items in Study 2**.

**Item**	**1**	**2**	**3**
I deliberately hid how much TIME I spent gambling from my family and/or friends.	**0.88**	0.02	−0.04
I deliberately hid how much MONEY I spent gambling from my family and/or friends.	**0.86**	0.01	−0.03
I experienced unwanted thoughts about my gambling when I WASN'T playing.	**0.77**	−0.03	0.08
I felt my gambling was out of control.	**0.74**	−0.01	0.10
I gambled to forget about problems in my life.	**0.73**	−0.01	0.02
I only spent time gambling that I could afford to spend.	0.08	**0.76**	−0.11
I only gambled with money that I could afford to lose.	0.00	**0.72**	−0.15
I considered the amount of MONEY I was willing to lose BEFORE I gambled.	−0.04	**0.67**	0.00
I considered the amount of TIME I was willing to spend BEFORE I gambled.	0.05	**0.61**	0.07
^**^I gambled just for entertainment.	−0.15	**0.55**	0.26
^**^I spent more MONEY gambling than I wanted.	−0.01	−0.01	**0.88**
^**^I spent more TIME gambling than I wanted.	0.01	0.07	**0.85**
^**^I used an ATM to take out money to CONTINUE gambling.	0.12	0.01	**0.66**

** *Items were culled following analysis. PPS, Positive Play Scale. Values in bold represent a moderate-to-large loading on each component*.

**Table 2:** **Rotated factor loadings from a principal component analysis of the PPS belief items in Study 2**.

**Item**	**1**	**2**	**3**
I should be able to walk away from gambling at any time.	**0.69**	−0.07	0.05
I should be aware of how much MONEY I spend when I gamble.	**0.69**	0.00	0.18
It is my responsibility to spend only money that I can afford to lose.	**0.69**	0.00	−0.02
I should only consider gambling when I have enough money to cover all my bills.	**0.67**	0.02	−0.02
I should only view my gambling as a form of entertainment.	**0.61**	−0.00	−0.10
^**^To try to win back my losses is a bad idea.	**0.57**	0.00	−0.04
^**^I would seek help if I felt I was losing control over my gambling behavior.	**0.47**	−0.03	0.13
^**^Each time I play a particular game, my chances of winning or losing are always the same regardless of whether I won or lost in the previous game.	**0.45**	−0.12	0.00
^**^The odds of winning are mostly against me when I gamble (i.e., I will eventually lose more than I win).	**0.35**	0.01	0.23
Gambling is a good way for me to make money.	−0.12	**0.69**	0.09
My chances of winning improve after I have lost.	−0.13	**0.64**	0.07
If I gamble frequently, it will help me to win more than I lose.	−0.09	**0.61**	0.12
It is fine for me to gamble to forget my problems.	0.06	**0.60**	−0.16
^**^Sometimes, I can predict the outcome of a gamble.	−0.02	**0.58**	0.10
^**^My gambling can make me feel important.	−0.14	**0.57**	0.04
^**^Having good luck is a valid reason for me to continue gambling.	0.17	**0.55**	−0.13
^**^It is fine for me to take money out of an ATM to CONTINUE gambling.	0.32	**0.44**	−0.41
^**^It is important to control how much TIME I spend gambling.	−0.04	0.06	**0.87**
^**^I should be aware of how much TIME I spend when I gamble.	0.17	0.01	**0.67**
^**^It is important to control how much MONEY I spend gambling.	0.31	0.06	**0.53**

** *Items were culled following analysis. PPS, Positive Play Scale. Values in bold represent a moderate-to-large loading on each component*.

## Study 2: factor structure assessment

In Study 2, we evaluated the psychometric properties of the PPS items developed in Study 1. To that end, we recruited a large sample of players to complete the PPS in order to determine its factor structure. Players also completed measures of gambling frequency, problem gambling severity index (PGSI: Ferris and Wynne, 2001), and gambling beliefs questionnaire (GBQ: Steenbergh et al., 2002) to assess the construct validity of the PPS. Because people who score higher on the PPS should be more likely to engage in RG, they should also be less likely to have gambling related problems, and have less erroneous beliefs about gambling. Thus, we hypothesized that players with higher scores on the PPS would be less likely to show symptoms of gambling problems and report less irrational cognitions.

### Method

#### Participants and procedure

A third party survey company (Vision Critical) recruited 1,551 customers of the British Columbia Lottery Corporation^1^ via email to complete an online survey about their gambling beliefs and behaviors. Participation was restricted to “regular players” (i.e., people who gambled at least once in the preceding month). Recruitment was based upon quota sampling, such that sex composition was relatively equivalent (male = 847, female = 704) and there was equal representation from players who primarily played lottery games purchased at a retailer (*n* = 303), casino games played in a casino (*n* = 413), slot-machine and table game players who were members of a loyalty program (*n* = 408), and online players (*n* = 427). They ranged in age from 19 to 65+ and most (58.4%) were between the ages of 55 and 65+. All participants were compensated $1 for completing the survey.

Ethical review and approval was not required for this study as per the institutional and national requirements. All participants were responding to a survey that was part of a prior customer agreement with the British Columbia Lottery Corporation, concerning the provision of feedback related to gambling beliefs and behavior. Nevertheless, participants were fully informed about the nature of the study, provided their written consent to take part and were free to withdraw at any point. All data was anonymized and the study was carried out in accordance with the British Columbia Freedom of Information and Protection of Privacy Act.

#### Measures

Participants completed the PPS items developed in Study 1 (see Tables 1, 2). Participants responded to each PPS belief item using a response scale anchored at 1 (*strongly disagree*) and 7 (*strongly agree*). For the PPS behavior items, participants responded using a response scale anchored at 1 (*never*) and 7 (*always*). Participants also completed the following two questionnaires:

##### Disordered gambling severity

The extent to which participants have gambling problems was assessed using the PGSI (Ferris and Wynne, 2001). The PGSI consists of nine items that measure the extent of problem gambling behaviors (e.g., “Have you gone back another day to try and win back the money you lost?”) and the consequences of engaging in problem gambling behaviors (e.g., “Has your gambling caused any financial problems for you or your household?”). Participants responded by indicating how frequently they engaged in problem gambling behaviors and experienced consequences due to their gambling behavior over the last 12 months. Responses were anchored at 0 (*never*) and 3 (*almost always*). Responses were summed such that higher scores indicated greater disordered gambling severity (α = 0.91).

##### Gambling beliefs

Participants' beliefs about gambling were assessed using the GBQ (Steenbergh et al., 2002). The GBQ consists of 21 items that are divided into two subscales. The first subscale consists of eight items that measure control beliefs about gambling (e.g., “My knowledge and skill in gambling contribute to the likelihood that I will make money”). The second subscale consists of 13 items that measure beliefs in luck (e.g., “Where I get money to gamble doesn't matter because I will win and pay it back”). Participants responded to each item using a response scale with endpoints 1 (*strongly disagree*) and 7 (*strongly agree*). Items for each subscale were averaged such that they reflected greater beliefs in control over gambling outcomes (α = 0.82) and greater beliefs in luck (α = 0.91).

##### Gambling frequency

Participants indicated the extent to which they engaged in the following land-based gambling activities: lottery draw games, scratch-cards, sports betting, bingo, electronic games (e.g., slot machines, puzzle games), casino style card games (e.g., poker, blackjack), and Casino style table games (e.g., roulette, craps). Participants also reported their frequency of playing according to different channels of access (e.g., online, a land-based casino, bingo hall, lottery ticket booth, grocery store, gas station). Participants reported their level of engagement using a response scale with endpoints 1 (*never*) and 7 (*more than once a week*).

### Results

#### Structure of PPS

PCA with promax rotation was conducted on the PPS. For the 13 PPS behavior items, three components were extracted each with an eigenvalue >1. The first component had an eigenvalue of 4.85 and accounted for 37.36% of the variance. The second component had an eigenvalue of 1.86 and accounted for an additional 14.32% of the variance. The third component had an eigenvalue of 1.03 and accounted for an additional 7.99% of the variance. Importantly, the scree test and parallel analysis (involving 5,000 resamples from the data)—a statistical procedure to determine the number of components to retain from PCAs (O'Connor, 2000)—both indicated that the optimal solution would be to retain only the first two components. The rotated loadings are reported in Table 1. For the first component, all five items had loadings >0.60 and thus were averaged to form a scale (α = 0.87). For the second component, four of the five items had loadings >0.60 and thus these four items were averaged to form a scale (α = 0.67). All remaining items (see Table 2) were excluded from subsequent analyses.

Likewise, for the 20 PPS belief items, results of the PCA indicated that three components were extracted each with an eigenvalue >1. The first component had an eigenvalue of 5.08 and accounted for 25.42% of the variance. The second component had an eigenvalue of 2.14 and accounted for an additional 10.73% of the variance. The third component had an eigenvalue of 1.29 and accounted for an additional 6.4% of the variance. Importantly, the scree test and parallel analysis both indicated that the optimal solution would be to retain only the first two components. The rotated factor loadings are presented in Table 2. For the first component, five of the nine items had loadings >0.60 and thus these items were averaged to form a scale (α = 0.74). For the second component, four of the five items had loadings >0.60 and thus these four items were averaged to form a scale (α = 0.66). All remaining items (see Table 2) were excluded from subsequent analyses.

### Descriptive statistics and bivariate analyses

Descriptive statistics and correlations for all variables in Study 1 are reported in Table 3. The magnitude of the intercorrelations between the four PPS subscales were small-to-moderate. This supports the view that four PPS subscales are related, but distinct, constructs. Importantly, as expected, the magnitude of the correlations between the four PPS subscales on the one hand and the PGSI and GBQ were all negative (see Table 3). As for gambling frequency, the four PPS subscales were either not associated with gambling frequency or had small-to-moderate negative relationships with frequency of play for particular games (see Table 3).^2^

**Table 3:** **Descriptive statistics and correlations between PPS subscales and other variables in Study 2**.

	**1**	**2**	**3**	**4**	**5**	**6**	**7**	**8**	**9**	**10**	**11**	**12**	**13**	**14**
1. Honesty and control	−													
2. Pre-commitment	0.28^**^	−												
3. Personal responsibility	0.29^**^	0.43^**^	−											
4. Gambling literacy	0.44^**^	0.20^**^	0.33^**^	−										
5. PGSI	−0.67^**^	−0.31^**^	−0.26^**^	−0.38^**^	−									
6. GBQ-luck	−0.44^**^	−0.18^**^	−0.31^**^	−0.61^**^	0.46^**^	−								
7. GBQ-Control	−0.28^**^	−0.09^**^	−0.20^**^	−0.47^**^	0.32^**^	0.69^**^	−							
8. Lottery draw games	−0.04	−0.05^*^	−0.03	−0.09^**^	0.11^**^	0.09^**^	0.06^*^	−						
9. Scratch-cards	−0.08^**^	−0.03	−0.02	−0.11^**^	0.13^**^	0.11^**^	0.10^**^	0.31^**^	−					
10. Sports betting	−0.14^**^	−0.02	−0.10^**^	−0.19^**^	0.17^**^	0.22^**^	0.27^**^	0.05^*^	0.11^**^	−				
11. Bingo	−0.06^**^	−0.02	−0.07	−0.09^**^	0.08^**^	0.11^**^	0.07^**^	0.12^**^	0.22^**^	0.25^**^	−			
12. Electronic games	−0.20^**^	−0.05^*^	−0.01	−0.11^**^	0.25^**^	0.13^**^	0.09^**^	0.20^**^	0.30^**^	0.08^**^	0.24^**^	−		
13. Casino card games	−0.15^**^	−0.03	−0.08^**^	−0.19^**^	0.21^**^	0.25^**^	0.30^**^	0.14^**^	0.20^**^	0.34^**^	0.27^**^	0.31^**^	−	
14. Casino table games	−0.18^**^	−0.09^*^	−0.12^**^	−0.17^**^	0.24^**^	0.26^**^	0.24^**^	0.13^**^	0.18^**^	0.36^**^	0.30^**^	0.27^**^	0.61^**^	−
*M*	6.63	5.76	6.45	6.42	0.97	19.78	16.88	2.39	2.00	1.14	1.13	1.74	1.24	1.15
*SD*	0.88	1.28	0.85	0.85	2.63	10.47	8.78	1.01	0.95	0.54	0.51	1.00	0.67	0.53

*PPS, Positive Play Scale; PGSI, Problem Gambling Severity Index; GBQ, Gambling Beliefs Questionnaire*.

* p < 0.05;

** *p < 0.01*.

*N = 1551*.

### Discussion

Results of Study 2 showed that RG can be measured empirically and is composed of four related, but distinct subscales: *Honesty and Control, Pre-commitment, Personal Responsibility*, and *Gambling Literacy*. Findings showed that most players in the sample scored fairly high on each subscale. This is to be expected as most players of gambling type games will not have, or be at risk for, developing a gambling problem. The PPS subscales were moderately and negatively associated with disordered gambling severity and erroneous gambling beliefs. However, the magnitude of the associations were not overwhelming, which suggests that the PPS is tapping into some similar items as the PGSI and GBQ, but overall it is measuring different constructs. That is, the PPS is not just measuring the absence of problematic gambling behavior and beliefs, but instead appears to be measuring the concept of positive play (i.e., RG as manifested by the player). This supports and extends the findings of recent studies that suggested RG is more than just an absence of gambling related problems or erroneous beliefs (e.g., Wood and Griffiths, 2015; Hing et al., 2016). Overall, frequency of play was not strongly correlated with the PPS subscales, indicating that RG is not simply a product of less frequent gambling. However, as shown in Table 3, frequent players of games that have been consistently linked to problem gambling (e.g., electronic games) showed lower PPS sub-scale scores, compared to players of games which are less commonly linked to problem gambling (e.g., lottery draw games). More frequent players of electronic games showed lower scores in relation to: *Honesty and Control, Pre-commitment*, and *Gambling Literacy* and higher scores on the PGSI. This may suggest that higher levels of problem gambling on electronic games could be more than just a function of the characteristics of the game, but may also be based on the individual players' level of responsible play. However, as this observation is correlational it could also be that problematic play on such games leads to less responsible behavior and beliefs. More research is needed to further explore the nature and direction of this relationship. This finding does suggest that RG strategy (for this sample of players) would benefit from focusing efforts to increase the scores of high-frequency electronic game players, in relation to the PPS sub-scales *Honesty and Control, Pre-commitment*, and *Gambling Literacy*.

Although we found the expected PPS factor structure and the PPS subscales were correlated with the PGSI and GBQ in the predicted directions, one potential limitation of the items was the high number of negatively worded items (e.g., Honesty and Control was only composed of negative items). Because the PPS is focused on positive play, we addressed this limitation in Study 3 by revising three of the negatively worded items to be positively framed.

## Study 3: factor validation and reliability assessment

The first aim of Study 3 was to replicate the factor structure observed in Study 2. We also revised the wording of three behavioral items so that that they were positively framed. For example, “I deliberately hid how much money I spent gambling from my family and friends” was revised to “I was honest with my family and friends about the amount of money I spent gambling.” All PPS items used in Study 3 are reported in Tables 4, 5. In addition, we assessed the test re-test reliability of the PPS by re-contacting a subset of participants approximately 1 month later to complete the PPS again.

**Table 4:** **Rotated factor loadings from a principal component analysis of the PPS behavior items in Study 3**.

	**9-items**			**7-items**	
**Item**	**1**	**2**	**3**	**1**	**2**
I considered the amount of TIME I was willing to spend BEFORE I gambled.	**0.94**	−0.17	−0.07	**0.93**	−0.20
I considered the amount of MONEY I was willing to lose BEFORE I gambled.	**0.89**	−0.03	0.02	**0.90**	−0.03
I only spent TIME gambling that I could afford to spend.	**0.69**	0.16	0.03	**0.69**	0.18
I only gambled with MONEY that I could afford to lose.	**0.67**	0.23	0.03	**0.67**	0.24
I was honest with my family and/or friends about the amount of TIME I spent gambling.	−0.03	0.**94**	−0.07	−0.06	**0.92**
I was honest with my family and/or friends about the amount of MONEY I spent gambling.	0.02	**0.91**	−0.05	0.01	**0.90**
I felt in control of my gambling behavior.	−0.03	**0.55**	0.21	0.02	**0.62**
^**^I gambled to forget about problems in my life.	−0.11	0.04	**0.88**	–	–
^**^I experienced bad thoughts about my gambling when I WASN'T playing.	0.12	−0.05	**0.82**	–	–

** *Excluded from the final scale. PPS, Positive Play Scale. Values in bold represent a moderate-to-large loading on each component*.

**Table 5:** **Rotated factor loadings from a principal component analysis of the PPS belief items in Study 3**.

**Item**	**1**	**2**	**3**
It's my responsibility to spend only money that I can afford to lose.	**0.92**	−0.03	−0.01
I should be aware of how much MONEY I spend when I gamble.	**0.88**	−0.01	0.02
I should only gamble when I have enough money to cover all my bills first.	**0.80**	−0.04	−0.06
I should be able to walk away from gambling at any time.	**0.78**	0.01	0.15
^**^I should only consider gambling as entertainment.	**0.55**	0.12	−0.16
If I gamble more often, it will help me to win more than I lose.	0.02	**0.85**	0.08
My chances of winning get better after I have lost.	0.01	**0.79**	0.01
Gambling is a good way to make money.	−0.02	**0.76**	−0.09
^**^Gambling to forget my problems is a bad idea.	−0.02	0.01	**0.97**

** *Excluded from the final scale. PPS, Positive Play Scale. Values in bold represent a moderate-to-large loading on each component*.

The second aim of Study 3 was to further examine the validity of the PPS through associations with constructs that have been linked to gambling in prior research. These constructs were impulsivity, the Big-Five personality traits, financially focused self-concept, financial dissatisfaction, and general self-efficacy. We expected that higher PPS scores would be associated with lower impulsivity. The reason is that impulsivity is a known risk factor for problem gambling (Blaszczynski and Nower, 2002). For the Big-Five, we expected that higher PPS scores would be associated with lower neuroticism and higher conscientiousness, and be unrelated to extraversion, agreeableness, and openness to new experiences. The reason is that disordered gamblers (relative to most regular players) are likely to be more neurotic as well as less conscientious (MacLaren et al., 2015). For financially focused self-concept and financial satisfaction, we expected higher PPS scores to be associated with lower financially focused self-concept and higher financial dissatisfaction. This is because players who are focused on financial success are more likely to have gambling problems (Tabri et al., 2017). Lastly, for general self-efficacy, we predicted that higher PPS scores would be associated with greater general self-efficacy. This is because people who have low control over their gambling behavior are likely to have low self-efficacy scores (Kaur et al., 2006).

### Method

#### Participants and procedure

A total of 443 community gambling players were recruited using the same third party survey company in Study 1 to complete an online survey about their gambling attitudes and behaviors. However, the data of 31 participants were excluded from the analyses because these participants failed one or more attention checks. Thus, the data of 412 (male = 225, female = 218) participants were included in the analyses Participants ranged in age from 19 to 65+ and most (59.4%) were <65 years of age.

A subset of participants (*N* = 149) were re-contacted 1 month later to complete the PPS a second time. Of the 149 participants who completed the PPS, the data of two participants were excluded because they failed one or more attention checks. Thus, the data of 147 (male = 72, female = 75) participants from the initial data collection of Study 3 were included in the follow-up analyses.

#### Measures

Participants completed a revised version of the PPS as well as the PGSI and GBQ used in Study 1. They also completed the following measures:

##### Ten-item personality inventory (TIPI; Gosling et al., 2003)

We used the TIPI to assess the Big-Five personality domains. Each personality domain was measured using two pairs of traits. For extraversion, the traits were “extraverted, enthusiastic” and “reserved, quite” (reverse coded). For agreeableness, the traits were “sympathetic, warm” and “critical, quarrelsome” (reverse coded). For conscientiousness, the traits were “dependable, self-disciplined” and “disorganized, careless” (reverse coded). For neuroticism, the traits were “anxious, easily upset” and “calm, emotionally stable” (reverse coded). For openness to new experiences, the traits were “open to new experiences, complex” and “conventional, uncreative” (reverse coded). Participants rated the extent to which each pair of traits applies to them. Responses were anchored at 1 (*strongly disagree*) and 7 (*strongly agree*). We averaged responses such that higher scores reflect greater neuroticism, extraversion, agreeableness, conscientiousness, and openness to new experiences.

##### Barratt Impulsiveness Scale (BIS; Spinella, 2007)

The short form 15-item BIS was included to assess participants' degree of impulsivity. Like the original long form 30-item BIS, the 15-item short form BIS are equally distributed across three subscales that measure the extent to which people engage in non-planning (e.g., “I say things without thinking”), motor impulsivity (e.g., “I act on impulse”), and attentional impulsivity (e.g., “I am restless at lectures or talks”). Responses were anchored at 1 (*rarely/never*) and 4 (*almost always/always*). We averaged responses across subscales such that higher scores reflect greater impulsivity (α = 0.81).

##### Financially focused self-concept (FFS; Tabri et al., 2017)

The short form of the FFS was used in the present research. This questionnaire includes four items that measure the extent to which people's self-concept is focused on financial success. The four FFS items were “How I feel about myself is largely based on the amount of money I have,” “My moods are influenced by the amount of money I have,” “People will think less of me if I don't have a lot of money,” and “The opportunities that are available to me depend on the amount of money I have.” Participants' responses were anchored at 1 (*not at all*) and 5 (*extremely*). We averaged responses such that higher scores indicated greater financially focused self-concept (α = 0.77).

##### InCharge financial distress/financial well-being scale (IFDFW; Prawitz et al., 2006)

The IFDFW scale consists of eight items measuring the extent to which participants are satisfied with their financial situation. Items were “What do you feel is the level of your financial stress today?,” “How stressed do you feel about your personal finances in general?,” “How often does this happen to you? You want to go out to eat, go to a movie or do something else and don't go because you can't afford to?,” “How frequently do you find yourself just getting by financially and living paycheck to paycheck?,” “How satisfied are you with your present financial situation,” “How often do you worry about being able to meet normal monthly living expenses?,” and “How confident are you that you could find the money to pay for a financial emergency that costs about $1,000?” Participants responded using a scale with anchor labels that varied by item. For the first two items, the anchors were *overwhelming stress* (1) and *no stress at all* (10). For the third and fourth items, the anchors were *all the time* (1) and *never* (10). For the fifth item, the anchors were *feel overwhelmed* (1) and *feel comfortable* (10). For the sixth item, the anchors were *complete dissatisfaction* (1) and *complete satisfaction* (10). For the seventh item, the anchors were *worry all the time* (1) and *never worry* (10). For the eighth item, the anchors were *no confidence* (1) and *high confidence* (10). Consistent with Prawitz et al. (2006), we averaged across items such that higher scores indicate great financial satisfaction (α = 0.94).

##### General self-efficacy (GSE; Schwarzer and Jerusalem, 1995)

The 10-item GSE was used to measure the extent to which participants' perceive that they have a general sense of self-efficacy to face difficulties (e.g., “I can always manage to solve difficult problems if I try hard enough.”) and attain goals (e.g., “It is easy for me to stick to my aims and accomplish my goals.”). Responses were anchored at 1 (*not at all true*) and 4 (*exactly true*). We averaged responses such that higher scores reflect greater perceived general self-efficacy (α = 0.85).

### Results

#### Structure of the PPS

PCA with promax rotation on the nine PPS behavior items (see Table 4) revealed three components with an eigenvalue >1. The first component had an eigenvalue of 4.06 and accounted for 45.19% of the variance. The second component had an eigenvalue of 1.17 and accounted for an additional 13.05% of the variance. The third component had an eigenvalue of 1.15 and accounted for an additional 12.82% of the variance. However, the scree test and parallel analysis both indicated that the optimal solution would be to retain only the first two components. The rotated loadings are reported in Table 4. As such, we repeated the PCA after omitting the two items that loaded on the third component. Importantly, we observed the same two components from the PCA with seven items. Moreover, the scree test and parallel analysis both indicated that the optimal solution would be to retain both of these components. The four pre-commitment items that loaded on the first component had loadings >0.60 and thus were averaged to form a scale (*M* = 6.52, *SD* = 0.94; α = 0.84). Likewise, the loadings for the three honesty and control items that loaded on the second component were >0.60 and thus were averaged to form a scale (*M* = 6.45, *SD* = 1.13; α = 0.76).

Likewise, for the nine PPS belief items (see Table 5), results of the PCA indicated that three components were extracted each with an eigenvalue >1. The first component had an eigenvalue of 3.40 and accounted for 37.83% of the variance. The second component had an eigenvalue of 1.79 and accounted for an additional 19.91% of the variance. The third component had an eigenvalue of 1.01 and accounted for an additional 11.31% of the variance. Importantly, the scree test and parallel analysis both indicated that the optimal solution would be to retain only the first two components. The rotated factor loadings are presented in Table 5. The five personal responsibility items that loaded on the first component had loadings >0.50 and thus were averaged to form a scale (*M* = 6.79, *SD* = 0.55; α = 0.82). Likewise, the three gambling literacy items that loaded on the second component had loadings >0.75 and thus were averaged to form a scale (*M* = 6.46, *SD* = 0.90; α = 0.73). The remaining item that loaded on the third component was excluded from further analyses (see Table 5). The final scale contained seven items related to behavior and seven items related to beliefs.

#### Descriptive statistics and bivariate analyses

Descriptive statistics and correlations between the PPS and all other variables are reported in Table 5. As in Study 2, higher scores on each of the PPS subscales were negatively correlated with scores on the PGSI as well as with scores on the GBQ luck and control subscales. In addition, higher scores on the PPS subscales were each associated with greater conscientiousness, lower neuroticism, lower impulsivity, greater financial satisfaction, greater general self-efficacy, and lower FFS. The magnitude of these associations were weak-to-moderate (see Table 6).

**Table 6:** **Descriptive correlations between PPS subscales and all other factors measured in Study 3**.

	***M* (*SD*)**	**Honesty and control**	**Pre-commitment**	**Personal responsibility**	**Gambling literacy**
PGSI	0.17 (2.23)	−0.32^**^	−0.38^**^	−0.03	−0.15^**^
GBQ-luck	19.81 (9.43)	−0.21^**^	−0.32^**^	−0.25^**^	−0.58^**^
GBQ-Control	17.23 (8.70)	−0.03	−0.16^**^	−0.13^**^	−0.39^**^
Neuroticism	2.69 (1.21)	−0.07	−0.14^**^	−0.11^*^	−0.21^**^
Openness	4.87 (1.18)	−0.02	0.08	0.06	0.02
Conscientiousness	5.72 (1.03)	0.17^**^	0.22^**^	0.21^**^	0.14^**^
Extraversion	3.97 (1.41)	−0.06	0.01	0.05	−0.04
Agreeableness	5.27 (1.10)	0.08	0.09	0.05	0.09
BIS	1.92 (0.40)	−0.21^**^	−0.28^**^	−0.23^**^	−0.17^**^
General self-efficacy	3.20 (0.37)	0.09^†^	0.15^**^	0.12^*^	0.09
Financial satisfaction	7.57 (2.10)	0.14^**^	0.25^**^	0.13^**^	0.29^**^
FFS	1.73 (0.60)	−0.19^**^	−0.26^**^	−0.09^*^	−0.24^**^

*PPS, Positive Play Scale; PGSI, Problem Gambling Severity Index; GBQ, Gambling Beliefs Questionnaire; BIS, Barratt Impulsivity Scale; FFS, Financially Focused Self-Concept*.

† p = 0.05;

* p < 0.05;

** *p < 0.01*.

*N = 412*.

#### Test re-test reliability of the PPS

The same PPS subscales identified with the total sample of Study 3 were computed for the subsample that completed the PPS 1 month later. The magnitude of the test re-test correlations for the pre-commitment (*r* = 0.62, *p* < 0.001), honesty and control (*r* = 0.39, *p* < 0.001), personal responsibility (*r* = 0.37, *p* < 0.001), and gambling literacy (*r* = 0.43, *p* < 0.001) PPS subscales were moderate to strong. These results indicate that participants' relative rankings on the PPS subscales tended to be stable over time.

### Discussion

The results of Study 3 replicated the overall structure of the PPS for seven of the nine behavior items and seven of the nine belief items. In addition, results of Study 3 demonstrated that the PPS subscales had good test-retest reliability, which suggests that the PPS is reliable over time. Study 3 also replicated the correlations between the PPS on the one hand and the PGSI and GBQ observed in Study 2, which further supports the reliability of the PPS. As well, findings from Study 3 showed that the PPS subscales were each associated with greater conscientiousness, lower neuroticism, lower impulsivity, greater financial satisfaction, greater general self-efficacy, and lower FFS. These findings suggest that the PPS is measuring a construct that is linked to an overall sense of well-being in terms of reported behaviors and psychological constructs that we might reasonably expect to accompany RG, as defined in our working definition.

## General discussion

Like many industries that can produce harms, it is often challenging to disentangle the relative contributions of the gambling industry to wider public health outcomes. While reduction of harms may be an overall public health goal, responsible gambling programs are only part of a larger solution that also includes treatment programs, public education, and other policy decisions around availability and support.

The current research developed and assessed the psychometric properties of a new PPS. Consistent with what is known about RG, the scale assesses two unique components: players' positive (or responsible) beliefs about gambling and players' positive (or responsible) gambling behaviors. Importantly, these components produced reliable and valid data in two studies utilizing the customers of a provincial Canadian gambling operator.

Using the PPS should help to optimize gambling operators' RG strategy by objectively measuring the effectiveness of programs. Consequently, less money and resources are wasted on strategies that are ineffective, while effective elements can be more fully supported. For example, if a gambling operator finds that a sample of slot machine players are scoring low on the pre-commitment sub-scale, they could focus future RG efforts on promoting the use of limit setting tools. The PPS should be particularly useful when introducing new RG initiatives (e.g., an education campaign aimed at dispelling gambling fallacies). To this end, the PPS could be used in pre-post evaluations to identify any positive or negative changes following the implementation. That is, does the new RG initiative result in a measurable increase in PPS scores for the player population. Furthermore, the PPS offers the opportunity to more effectively measure beliefs and behaviors by providing a means by which to measure changes toward the positive end of the gambling spectrum, which should include the vast majority of players.

The PPS could also aid RG strategies to become more segmented, by identifying the approaches that work best with different player demographics and according to game types. In their study of product harm and consumer vulnerability to marketing, Smith and Cooper-Martin (1997) suggest that firms are better served by focusing on product harm reduction rather than on business development with lower-risk groups. They also find that policymakers should focus on vulnerabilities in clearly defined groups of consumers. The PPS better enables both of these objectives. For example, some RG approaches may work best with younger players, with women, with slot-machine players etc. In other words, the PPS could help tailor RG programs to the individual consumer (e.g., game of choice, age, gender) based on empirical evidence.

The PPS should facilitate a more positive (carrot-based) approach to behavior change, by examining and encouraging positive patterns of play amongst the majority of players. Hence, enabling the focus of RG strategy to be refined to include the much wider base of players whose behavior and beliefs are largely positive, but who may benefit from information and initiatives that are aimed to promote elements of RG that could be further strengthened (e.g., encourage engagement with limit-setting tools). This contrasts with the largely negative (stick-based) approach that currently underpins most RG strategies. A more positive approach is likely to resonate well with players (i.e., “this is how to maximize positive playing experiences”), rather than negative messages that infer problem gambling (i.e., “if you don't play responsibly you will experience problems”; Wood and Griffiths, 2015). Additionally, a positive play approach should attenuate the stigma typically associated with extant stick-based RG initiatives (i.e., “these strategies are for people with problems”). In doing so, non-problematic players may be more likely to relate to and engage with RG strategies, thus showing utility for problem gambling prevention.

Moreover, including the PPS in public health research like gambling prevalence studies, offers the opportunity to gain much more detailed information about the overall level of healthy gambling behavior amongst players. This would be a significant shift in perspective. Indeed, both gambling regulations and RG policies are frequently informed through an examination of problematic gambling (typically a hundred or so players from a total sample of thousands). Furthermore, by focusing on the much larger sample of positive players, more subtle differences in responsible playing behaviors and beliefs should be observed. As such, it becomes more readily possible to focus harm minimization initiatives toward those players with much less severe indicators of risky behavior and beliefs (i.e., lower scores on the PPS). The likelihood of successfully changing erroneous gambling beliefs and risky behaviors, should be increased when they are identified at an earlier stage. Consequently, we argue that lighter touch interventions could be employed that are more palatable to less responsible (but not necessarily problematic) players. This is because such customers may disregard interventions that appear to be aimed at players with more severe gambling related issues. For example, “nudging” people with milder health related issues has been shown to be more effective at the population level, than trying to intervene with just those people who have much more serious health issues to deal with Maretau et al. (2011). Nudging is just the kind of low intensity approach that would be suitable to use with the majority of players who are gambling as a means of entertainment.

We also contend that asking players to consider their positive playing beliefs and behaviors would be much less insensitive than asking them to complete problem gambling screens which focus on socially deviant negatively framed behaviors (stealing, lying, deception etc.). This is important because gambling is a leisure activity that players undertake for enjoyment. If most players are to be encouraged to play responsibly and to better understand their own gambling behaviors and beliefs, then the approach toward studying and subsequently promoting RG should arguably be more focused on positive play than gambling related problems. Such a positive approach may not just encourage RG engagement; it may also help counter the perception that RG is just for people with problems.

### Limitations and future directions

It would be fruitful for future research to replicate the structure of the PPS in a new sample using a confirmatory approach (e.g., Confirmatory Factor Analysis). This was not undertaken in the present studies because the PPS items were not identical across Studies 2 and 3. In Study 2, we tested the first iteration of the PPS. In Study 3, we revised the wording of some PPS items and cut a few items. For this reason, we opted to replicate the structure of the PPS using the same statistical method in both studies (PCA and parallel analysis).

Another possible limitation of the current research concerns the external validity of our findings. While the PPS was developed using players of many different products, the sample was from a single gambling jurisdiction (British Columbia). As such, the applicability of the PPS may be geographically limited. With that said, there is no reason to believe players in British Columbia are any different than players in the rest of Canada (or beyond). Future application of the PPS in other jurisdictions will help to test this notion further. Participants in the study were somewhat self-selected in that they had previously agreed to take part in research studies. Therefore, we might question how representative they were of players in British Columbia. Of course, to some extent all survey respondents are self-selecting, in that they accept or refuse a request to take part in the study. Again, this notion will be further elucidated through utilizing the PPS with additional samples. We would also argue, that what the study lacked in terms of the representativeness of players generally, it makes up for in terms of wider ecological validity by utilizing actual players, reporting on their real-life gambling behaviors and beliefs.

In interpreting the results of the PPS, an obvious question would be “what are the cut-offs to indicate high, medium and low levels of RG?” Currently there are no other similar scales by which to make comparisons and only one sample has so far been tested. As such, it is not yet possible to identify cut-offs. Over time as more samples are tested and more benchmark data is accumulated, then it should be possible to begin to designate meaningful cut-off scores. The inclusion of the PPS in prevalence studies would help to hasten this outcome. In the meantime, data gathered in a single population can be used to set benchmarks for later comparisons over time as well as to identify differences in the level of RG between various player segments.

Another possible limitation is that the PPS is based on self-reported behaviors and beliefs. There is always a risk that what people report and what they do are not always the same. To minimize this, participants were guaranteed total anonymity to help reduce any tendency to present themselves in a positive light. The overall level of problem gambling that was identified in the main sample (3.5% PGSI 8+) was somewhat higher than the 0.7% PG rate identified in the last BC prevalence study (Malatest et al., 2014). However, the sample in this study was comprised solely of regular gambling players, whereas prevalence studies include non-gamblers, and therefore the level of PGs identified is likely broadly representative of levels of PG more generally. Furthermore, it is worth noting that the vast majority of scales measuring health–related behaviors, beliefs/cognitions (e.g., PGSI, GBQ etc.) are also developed using self-report data and have been shown to be useful and valid tools. Nevertheless, in future studies it would be worthwhile comparing the reported behaviors of players with their actual player data, something that is now more of a possibility with the advent of player-cards and online player accounts.

Finally, the sensitivity of the PPS for measuring changes over time is yet to be tested. An examination of different player segments (e.g., lottery players vs. slot machine players) did show a reasonable variation between mean scores on the PPS sub-scale scores. This would suggest that the PPS is not being restricted by ceiling effects (i.e., everyone scoring high) or floor effects (i.e., everyone scoring low). Nevertheless, it will be necessary to have the same or a matched sample complete the scale again at a later date to observe any changes in scores over time. Ideally, this would take place following the implementation of a new RG initiative (e.g., an RG awareness campaign) so that there is a likelihood that changes would be evident. However, subsequent analysis should be mindful of the extent to which little change could be a result of the success (or lack of) of the RG initiative rather than the sensitivity of the PPS. The inclusion of additional pre and post measures (e.g., player interviews) should help to illuminate such findings.

## Conclusion

In the field of gambling studies, little or no empirical research has examined RG from the player's perspective. The current research addressed this gap in the literature by developing a coherent definition of RG as well as a novel tool to measure RG beliefs and behaviors. We demonstrated in two large samples of gambling players that the PPS is both a valid and reliable tool that is related to but distinct from problem gambling behaviors and beliefs. The current research provides a new approach for understanding and objectively measuring RG, that can be used to assess the efficacy of RG strategies that are designed to promote positive and responsible play amongst players.

## Ethics statement

All procedures involving human participants were performed in accordance with the 1964 Helsinki declaration and its later amendments. In addition, the study was carried out in accordance with the British Columbia Freedom of Information and Protection of Privacy Act.

## Author contributions

RW was the Principal Investigator for the study and contributed to the conceptual design, literature review, procedures, analysis, results, and discussion. MW, NT, and KP contributed toward the conceptual design, literature review, procedures, analysis, results and discussion.

## Funding

This study was funded by the British Columbia Lottery Corporation.

### Conflict of interest statement

RW has conducted previous consultancy work for BCLC. KP currently works for BCLC. In conducting the study, we were given full consent to investigate, analyze and report all findings that the authors perceived to be relevant. The aims and outcomes of the project were mutually beneficial to both the authors and BCLC. That is, both wished to develop an effective scale for measuring the extent of positive beliefs and behavior within a population of players. Therefore, we do not consider that there was a conflict of interest between the authors of this manuscript and the funders of the research. The authors declare that the research was conducted in the absence of any commercial or financial relationships that could be construed as a potential conflict of interest.

## Footnotes

^1^The British Columbia Lottery Corporation is a government owned entity that conducts and manages all legal casino, lottery, and online gambling in the Province of British Columbia.

^2^The pattern of correlations between the PPS and gambling frequency, but for online games was similar.
